# A roadmap for using DHIS2 data to track progress in key health indicators in the Global South: experience from sub-saharan Africa

**DOI:** 10.1186/s12889-023-15979-z

**Published:** 2023-05-31

**Authors:** Andrea Farnham, Georg Loss, Isaac Lyatuu, Herminio Cossa, Alexandra V. Kulinkina, Mirko S. Winkler

**Affiliations:** 1 Swiss Tropical and Public Health Institute, Kreuzstrasse 2, 4123 Allschwil, Basel, Switzerland; 2 University of Basel, Basel, Switzerland; 3 Ifakara Health Institute, Dar es Salaam, Tanzania; 4 Manhiça Health Research Centre, Maputo, Mozambique

**Keywords:** DHIS2, Routine health management information system, Health systems

## Abstract

High quality health data as collected by health management information systems (HMIS) is an important building block of national health systems. District Health Information System 2 (DHIS2) software is an innovation in data management and monitoring for strengthening HMIS that has been widely implemented in low and middle-income countries in the last decade. However, analysts and decision-makers still face significant challenges in fully utilizing the capabilities of DHIS2 data to pursue national and international health agendas. We aimed to (i) identify the most relevant health indicators captured by DHIS2 for tracking progress towards the Sustainable Development goals in sub-Saharan African countries and (ii) present a clear roadmap for improving DHIS2 data quality and consistency, with a special focus on immediately actionable solutions. We identified that key indicators in child and maternal health (e.g. vaccine coverage, maternal deaths) are currently being tracked in the DHIS2 of most countries, while other indicators (e.g. HIV/AIDS) would benefit from streamlining the number of indicators collected and standardizing case definitions. Common data issues included unreliable denominators for calculation of incidence, differences in reporting among health facilities, and programmatic differences in data quality. We proposed solutions for many common data pitfalls at the analysis level, including standardized data cleaning pipelines, k-means clustering to identify high performing health facilities in terms of data quality, and imputation methods. While we focus on immediately actionable solutions for DHIS2 analysts, improvements at the point of data collection are the most rigorous. By investing in improving data quality and monitoring, countries can leverage the current global attention on health data to strengthen HMIS and progress towards national and international health priorities.

## Background

High quality health data, as collected by health management information systems (HMIS), are a key component of planning for utilization of health services, prevention and vaccination campaigns, and even evaluating national health programs [1, 2]. This pressing need for timely and high quality data has become abundantly clear during the coronavirus (COVID-19) pandemic [3]. Investment in HMIS has often languished, however, particularly in low and middle-income countries (LMICs), hobbling the ability of policymakers and governments to use their own data to inform decision-making and increasing reliance on often inaccurate or incomplete estimates of local health needs from international bodies [4, 5]. The need for these data became particularly pressing after the introduction of the United Nations’ 2030 Agenda for Sustainable Development and its associated Sustainable Development Goals (SDGs), which meant that tracking progress towards health and other development goals became more important than ever [5, 6].

District Health Information System 2 (DHIS2) software is a new solution to the siloed data collection and program-dominated reporting that have characterized HMIS in LMICs, as we have previously highlighted in the WHO Bulletin [7]. DHIS2 was developed by the Health Information Systems Programme, with support from the Norwegian Agency for Development Cooperation, the United States President’s Emergency Plan for AIDS Relief, the Global Fund to Fight AIDS, Tuberculosis and Malaria, the United Nations Children’s Fund, and the University of Oslo [8]. The open-source platform includes data validation, visualization and analysis tools, readily allowing for access and manipulation of health data at central and local levels [8]. In particular, the use of electronic forms enables data collection with built-in quality control measures. Since 2011, it has become the most popular HMIS platform, used in over 70 LMICs [8]. Several countries have reported improved data completeness and timeliness after implementing DHIS2 [9–13]. However, despite the important innovation that the platform offers, we showed in our previous viewpoint that thus far DHIS2 data have been underrepresented in the scientific literature [7]. Now, over a decade after the initial development of the DHIS2 software, and during a period of intense focus on pandemic health statistics reporting, is a key time to prioritize timely and high quality collection of the most important health indicators and promote more widespread scientific use of DHIS2 data.

As part of a larger research project called the Health Impact Assessment for Sustainable Development (HIA4SD), we aimed to access and analyze DHIS2 data in four countries in sub-Saharan Africa: Burkina Faso, Ghana, Mozambique, and Tanzania [14]. As we began working with the wider community of researchers and decision-makers using DHIS2, we realized that while much excellent work is being done with DHIS2 data, analysts are encountering similar barriers across many countries. Based on our real-world experiences using DHIS2 data to track key health indicators in and around large infrastructure projects, and conversations with experts in DHIS2 data, we propose solutions to these barriers. Our objectives are twofold: first, to identify the important health indicators captured by DHIS2 that are relevant for tracking progress towards the SDGs in sub-Saharan African countries; and second, to present a clear roadmap for improving DHIS2 data quality and consistency. While we do discuss systemic issues to be solved, our aim is most especially to provide analysts currently using DHIS2 data with immediately actionable solutions for the most prevalent data issues, allowing analysts to produce sound analyses using existing data for use by national-level decision-makers or publication in the scientific literature.

## Methods

As part of a larger research for development project (HIA4SD project; https://hia4sd.net) [14], we partnered with local health institutes in Burkina Faso, Ghana, Mozambique, and Tanzania. These institutes usually already had established contacts with the local Ministries of Health (MoH). In some places, our collaborators already had direct access to the national DHIS2 software and data. In others, a data sharing agreement for DHIS2 was established in the framework of the project and MoH analysts extracted the data and shared it with us in aggregate form, usually at the monthly and health facility level where possible. In all countries, we interacted almost exclusively with the central DHIS2 analysts at the MoH or our partner institutes. In addition, we reached out more informally to a larger network of DHIS2 analysts in many more Sub-Saharan African countries through the Swiss Tropical and Public Health Institute in order to expand our knowledge and understanding of the DHIS2 application. A Slack channel was established to promote discussion on these topics. After a period of informal discussion and synthesis, a comprehensive document encompassing the identified problems and solutions was created by the authors.

## What are the most important indicators that can realistically be captured by DHIS2 to harmonize across countries?

**Fig. 1:**
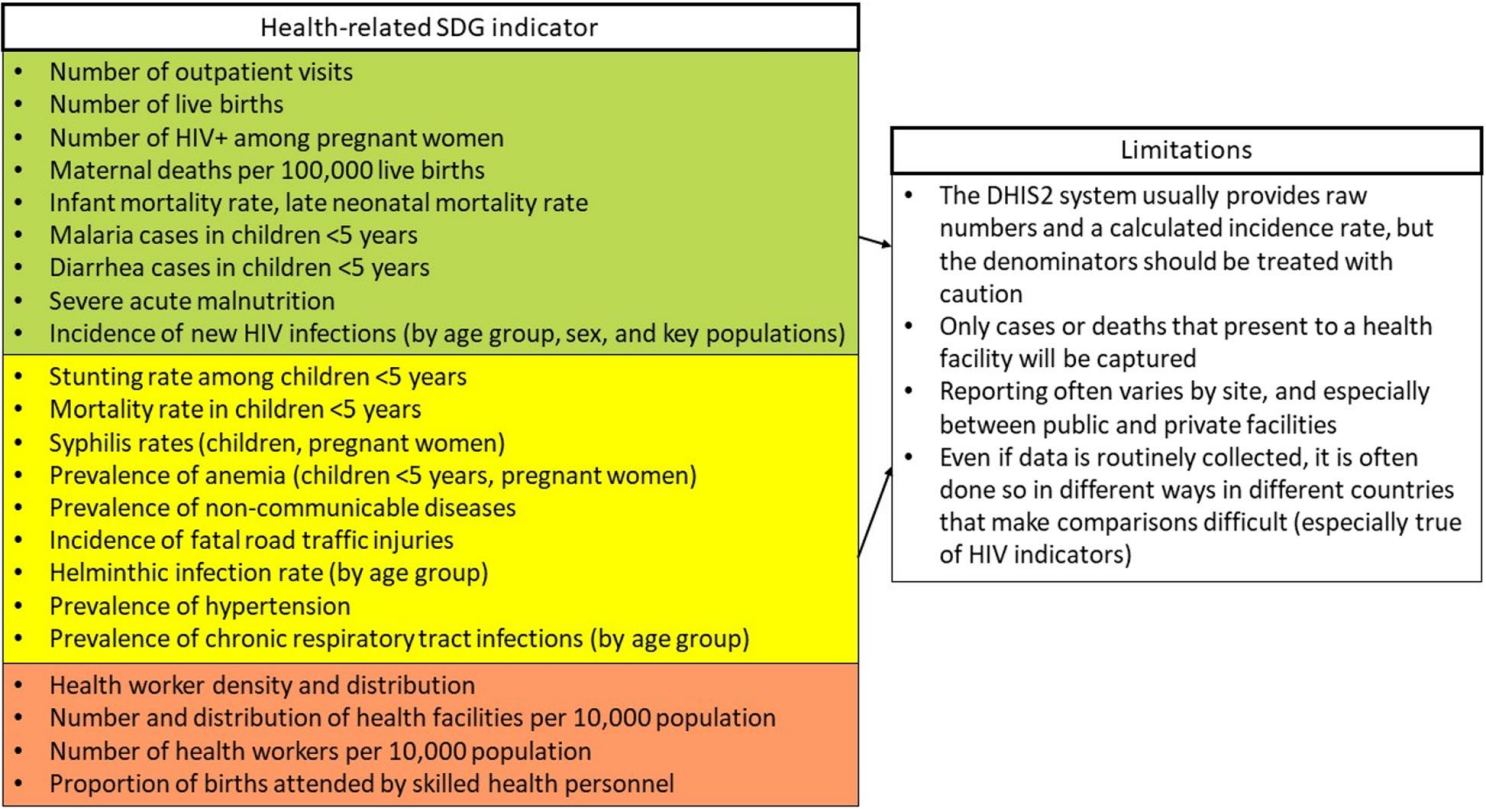
Health-related Sustainable Development Goal (SDG) indicators and our experience with the availability and limitations of DHIS2 data in four countries in Sub-Saharan Africa. Green corresponds to indicators that are routinely captured in most countries, yellow to indicators captured in some countries, and red represents indicators that are likely not suitable to be captured by DHIS2.

Each country has its own implementation of DHIS2 software [8], reflecting its own needs and priorities. However, we found some commonalities across all four study countries. Figure 1 presents a list of the health-related SDG indicators and our evaluation of whether they can be adequately captured by the DHIS2 system as it currently stands. In particular, maternal and child health outcomes are captured and reported almost universally, including maternal and child mortality in health facilities, infectious disease, antenatal care, and vaccination coverage. While these indicators are often standard across countries, the data quality varies by indicator and by country, and often even by health facility, as discussed further in the subsequent sections.

Other indicators, such as positive HIV cases, are widely captured and reported, but in a variety of different ways that inhibit comparison across time or countries. In fact, the huge variety of indicators around HIV testing makes it difficult to identify which variable to use as the definitive indicator for HIV case counts (example from one country of the seemingly similar indicators available: cases of HIV/AIDS, patients HIV positive, HIV positive test result). It is unclear whether each of these indicators refers to incident or prevalent cases, for example. Careful documentation of definitions and identification of which indicators are most important for national priorities should be readily available.

Almost universally, DHIS2 implementation would benefit greatly from standardized case definitions of the most important indicators for analysis, whether to track progress towards the SDGs, or to ensure that the specific programmatic concerns of each country are adequately tracked. This will require close collaboration between national policy makers at the MoH and governmental agencies, the DHIS2 implementation team, and staff from key national health programs. The proliferation of data indicators over time is a well-known problem, and regular database maintenance and elimination of unused indicators (after archiving any remaining data) should take place. Agreement on these issues will allow local data collectors to focus their time and resources on uploading the most important indicators at each site.

In general, certain key health indicators are not suitable to be captured in DHIS2 in its current form. In particular, mortality indicators are among the most difficult and inaccurate indicators in routine HMIS. Promising work in the global South has been done with conducting verbal autopsy to supplement the limited data available from HMIS [15]. In addition, data on health systems (e.g. number of health workers, capacity, services) has not often been captured within DHIS2, although these could also be a useful national metric for health. Instead, most countries rely on periodic implementations of the Service Availability and Readiness Assessment (SARA), a health facility assessment tool developed by the WHO. Better integration of these existing data collection systems with data from DHIS2 could be a powerful way to better track progress in health systems without overloading the HMIS.

### What are some actionable solutions for achieving better data quality and quantity in key health indicators?

As part of our study, we also became familiar with the process by which health data are collected, entered, and uploaded into the DHIS2 system, which was remarkably similar across countries. Most case reports are initially done on paper by local health staff at the facility, and then at the end of each month these case reports are collated into summary reports and entered into the software. Most countries have procedures for ensuring accuracy by comparing these summary reports with physical entries in the register books and what is entered into the DHIS2 software, often by trained staff that travel between health facilities. These procedures occur anywhere from monthly to once or twice per year, depending on the resources available. Fully digital data collection has traditionally not been seen as possible in many settings due to limited resources such as personnel, information technology (IT) infrastructure, internet connectivity and stable electricity, although case studies such as in Mali have shown that distribution of tablets for data collection can further improve this process [16]. While there are data quality assurance tools available within DHIS2 to check the accuracy of data entry, the degree to which these are used by local data entry staff is often limited. This similarity of data collection and processing across countries due to the similar structure of the health systems has the added advantage that identified solutions are likely to work in many settings.

DHIS2 data quality and quantity can be improved during four main time periods (Table 1). The first is to improve data collection in health facilities through more complete capture and recording of case reports on paper registers. The second is to improve the monthly tallying process during data aggregation. The third is to improve quality control during the data entry and upload process. The fourth is to correct for data shortcomings *post-hoc*, usually through statistical analysis techniques (e.g. imputing missing data). The periodic data quality assurance procedures carried out in most countries on a quarterly or annual basis at the health facilities represent an important opportunity to implement many of the mitigation activities suggested.

The most rigorous data improvement interventions occur on the level of data collection and entry; hence, we propose solutions to improve data collection processes ranging from digital staff training platforms to automated data reporting and quality control to performance-based funding (Table 1). In particular, regular trainings of data collectors and routine definition and documentation of key variables on the national level by each respective MoH would yield significant improvements in data quality and completeness. It is especially key to train and support the staff who conduct the monthly validation of DHIS2 data entry. Giving regular feedback and access to DHIS2 analysis tools to facility-level managers may also incentivize health facilities to optimize their own data collection processes. The World Health Organization (WHO) has collaborated with DHIS2 to produce standardized digital health toolkits [17], which could play a major role in developing and disseminating data standards across countries. Understanding and implementation of these toolkits should be of high priority for further improving data quality, especially for indicators like HIV where data collection remains unstandardized among countries. However, these solutions are also inevitably the most difficult and time and resource intensive. Therefore, we also propose solutions at the analysis level, with the goal of enabling analysts to utilize DHIS2 data in its current form.

Some problems, such as being able to differentiate between zero cases and missing data, seem to have a relatively straightforward technical fix. This issue was reported as a key frustration for many analysts working with DHIS2 data, and especially limits the ability of analysts working with national level data to use imputation methods to correct for missing data. Other problems, such as the large differences in data quality between different indicators, are often a result of differences in how data are collected by different programs and priorities of countries. The analyst can work around these differences by working with experts in the local health systems to identify and correctly define the appropriate indicators to use. One key takeaway of our research project is that strong partnerships with local data experts are absolutely crucial to utilizing the full potential of DHIS2 data.

**Table 1:** Systematic solutions for improving data quality and quantity from DHIS2, with a special focus on strategies for the analysis phase

Issue	Implication	Mitigation
**Time period 1: during data collection “on the ground”**		
Reporting varies by facility, especially between public and private facilities [18]	It is difficult to accurately capture regional incidence of cases when not all facilities are reporting. Aggregation of data at the regional and national level is meaningless if there are differences in reporting across regions.	- Various strategies to incentivize accurate reporting through performance-based funding (without linking funding amounts to case numbers) have been used successfully [19], although sustainability is a problem - Increase built-in functionality to capture and report data quality issues - Automating manual data collection, including integration with electronic medical records where possible - Improved training of staff responsible for monthly data entry and validation using inexpensive digital learning platforms [20, 21] *On the analysis level* - Instead of using data aggregated at the regional level, use reporting by individual facilities. Strategies such as k-means clustering have been used to use patterns in reporting to identify high and low performing health facilities [22]
Denominator data not always available	Calculating population-level rates is challenging; increases in case counts over time can be difficult to interpret without knowing changes in the local population.	- As part of routine data collection, estimates of the local catchment area population each health facility is serving could be updated yearly by local experts *On the analysis level* - When using aggregated data, census estimates could be used - Other datasets (e.g. WorldPop) could be utilized in cases where local population size is difficult to calculate
**Time periods 2–3: during the data entry and upload process**		
Large differences in quality between indicators	Generally, every indicator comes with its own set of specific issues and definitions, often determined by programmatic priorities; a unified approach to clean and analyze all indictors in a similar fashion is not possible	- By streamlining the number of required indicators to the most important ones and employing a dedicated DHIS2 staff member trained in data collection and reporting - Automating manual data collection, including integration with electronic medical records where possible - Using data validation rules during data entry - Automatic outlier detection functions *On the analysis level* - Identify and analyze only the most high quality indicators in a standardized process [23]
Both reports of “zero cases” and missing values are reported in the same way (on monthly level)	We cannot distinguish zero reported cases from missing values, introducing bias	- Adjust DHIS2 software to require separate zeroes or unknowns for complete reports *On the analysis level* - Reporting rates could be used to determine if report was filed; if so, empty cells could be assumed to be zero cases reported - Compare paper registry level data vs. DHIS2 level data (if available to analyst) - Use imputation methods on monthly data level if zero cases are rare
Total attendance by facility is sometimes reported through a different process than cases on the reporting sheets (i.e. not through the monthly tally sheet). Monthly reports may be missing total attendance, but report cases of individual conditions. Similarly, first and re-attendance recording can differ. The way that attendance numbers are collected is often only documented in a limited way.	Missingness of total attendance may differ from cases per month, and can lead to wrong denominator for rates; this can be missed by an analyst only looking at yearly data	- Make reporting attendance a standard part of reporting *On the analysis level* - Explore magnitude of the issue (using monthly level data) - Impute total attendance (on monthly level) - Explore other reliable denominators instead of case/attendance (see above) - Acquire registry level data (on facility/health program level)
Limited documentation	Lack of exact definitions of variables affects interpretation, comparability of variables across regions, generalization	- Responsible units (e.g., ministries of health) should define and document key variables together with local staff
**Time period 4: correcting for data limitations at the analysis phase**		
Extracting monthly level data at the facility for large areas such as country wide to do more complex analyses in external statistical software is very resource intensive and often not possible on a practical level	Limits any cleaning or analysis on monthly level; i.e., understanding the degree of missing data from individual health facilities is impossible to see from annual case counts only	- Use monthly level data for smaller areas only; imputation can then be used to extend the results to larger areas if necessary - Create finalized datasets for the most important monthly indicators for analysts to use at the end of each year
Data available with different periodicity (e.g. some monthly, some quarterly, some yearly)	Quality parameters for all indicators only possible on highest level (year) (often due to the fact that the appropriate population denominator is only available from the yearly census data)	- First assess quality for all indicators on highest level (year) - Then potentially re-extract data on monthly level for individual indicators where it is available
Data on some indicators available only from higher level health facilities and can therefore only be analyzed at the regional level (e.g. C-sections are often performed only at hospitals)	Some indicators reported at health facility level, others at district level; sometimes difficult to compare different indicators or assess data quality	- Discuss with local experts to understand where services are provided - Assess quality of these indicators only at the level of the health facility where they are provided
Reporting over time or region may be variable: changes over time and region are multi-causal (e.g. a decrease in malaria incidence may be due to decreases in reporting and/or a new malaria prevention program)	Increase/decrease of cases can be due to artefacts such as increased reporting rather than actual increase in cases	- Consult local experts to interpret any type of unexpected changes observed in data (e.g., spikes due to outbreaks; public health programs that increased reporting) - Adjust estimates of incidence by incorporating measures of local health-seeking behavior reported by other representative surveys (e.g., Demographic and Health Survey can be used to determine how many women accessed prenatal care on average in a region) [24] - Assess data quality by year (reporting rates)
Extraction process introduces variability in data (e.g., technical errors can produce different outputs for same query)	Quality of indicators and results are driven by extraction	- Standardize data cleaning process using semi-automatic procedures such as described in [23]
Diagnostic capabilities vary across facilities and regions (e.g. availability of PCR testing)	Underreporting in areas/facilities with lower diagnostic capabilities may lead to spurious associations derived from data	- Consult with local experts on each outcome/indicator, especially those requiring advanced diagnostic capabilities - Quantify extent of issue - Use of existing databases on health facility capability such as SARA to understand differing diagnostic capacity
Data aggregated on district level, although it is generated by point-based health facilities	Modifiable Areal Unit Problem effects (MAUP), a spatial statistical problem when point-based data are aggregated into districts [25]	- Use facility level data where possible - Focus on local spatial regression rather than global - Overlay aggregation method for disease mapping [26]

## Conclusions

Our experience working with DHIS2 data as part of the HIA4SD project indicates that many key health indicators (e.g. child and maternal health indicators) are already well captured by the platform; other indicators, such as HIV/AIDS incidence and prevalence, would benefit from more standardized case definitions and streamlining the number of indicators collected. While variations in facility level reporting, availability of denominator data, and differences in quality between different indicators remain a systemic problem, we have identified the above workarounds for these problems that should be shared more widely with the entire constellation of DHIS2 analysts and data users.

By focusing on improving a more limited number of indicators and resolving known data quality issues, countries utilizing DHIS2 software can dramatically improve their ability to monitor and evaluate progress towards national and international health targets. The global COVID-19 crisis has created a particular incentive and opportunity for LMICs to invest in their HMIS, potentially creating a lasting positive impact on local health capacities, ability to implement and evaluate new health programmes, and real-time monitoring of emergent health conditions. Some countries have already seen the benefits of these investments. During the pandemic, Côte d’Ivoire used its local implementation of DHIS2 to track COVID-19 rumors in real-time [27] and Bangladesh extended pandemic surveillance to collect cancer screening data [28]. These case studies should serve as examples of how LMICs can leverage the flexibility of DHIS2 software to advance their own priorities for their health systems. Small investments in DHIS2 training now could have immediate payoff for resolving the known data quality issues; for example, there is a widely available tool for data quality assurance available for DHIS2 [29] that appears to be underutilized at the country level [23]. The digital health toolkits that the WHO has developed in collaboration with DHIS2 teams should be utilized by countries to maximize data quality at all time periods and standardize data collection and reporting across countries, especially for key indicators such as HIV. A robust HMIS is an essential part of a strong health system and a key part of supporting evidence-based policy and decision-making, and should be both a national and international priority.

On the analyst level, there is no need to continually “reinvent the wheel” in terms of the data approaches to solve known data issues; instead, we propose that this perspective serve as a systematic catalogue of the data techniques that have been used thus far to improve the scientific quality of DHIS2 analyses. Our experience suggests that in-country analysts who are able to contextualize programmatic and technical changes that may affect data analysis and interpretation are indispensable to being able to use DHIS2 data, and should be recognized as such. Ideally, those tasked with analyzing and interpreting DHIS2 data should have access to a standardized list of data solutions such as we propose here, and an international network of analysts knowledgeable about how to work with DHIS2 data. Investment in the human resources around HMIS is no less important than the technical abilities that DHIS2 offers.

Several ideological challenges around using HMIS data to track progress towards the SDGs also remain, and are important to consider. First of all, there is still not a strong culture around data use by those developing, maintaining, and analyzing national level HMIS. Using HMIS data to generate evidence is largely restricted to minimal analysis of a large set of indicators, leading to a surface level understanding of the results. Related to this, most of the users routinely using DHIS2 to generate reports and analyses have minimal analytical skills and little incentive to delve deeply into the complexities of the DHIS2 database to answer more sophisticated questions. The HIA4SD study offers one template for how academic institutes can partner directly with MoH to use DHIS2 data to produce complex analyses and scientific publications. In terms of capacity building, we found partnerships to be particularly effective when the contact person at the MoH was partnered with the academic institute already during their training (e.g. when completing a Masters in Public Health). These types of partnerships have enormous potential in the longer-term to strengthen the culture around data use and analysis using national HMIS systems.

The international rollout of DHIS2 software over the past decade has offered clear opportunities for countries to own their data and lead improvements to national level HMIS. Nevertheless, the use of these data in the academic literature and by policy-makers has lagged. We believe that there is no time like the present to invest further in DHIS2 and bring HMIS in LMICs to the next level. Some fixes will require national level coordination, such as implementing more robust data quality measures and identifying and harmonizing indicators across the platform (e.g. through a core indicator classification system), while remaining sensitive to the needs of individual countries. Other improvements can be implemented at the analyst level, by standardizing data approaches and cleaning techniques across countries. Up-to-date and relevant HMIS data are clearly a current global priority, and countries using DHIS2 software can benefit today and in the future by maximizing its potential.

## Data Availability

Data sharing is not applicable to this article as no datasets were generated or analysed during the current study.

## Abbreviations

HMIS: Health Management Information Systems
LMIC: Low and middle-income countries
MoH: Ministry of Health
SDG: Sustainable Development Goal
DHIS2: District Health Information System 2
HIA4SD: Health Impact Assessment for Sustainable Development
